# Neural network-based surrogate model in postprocessing of topology optimized structures

**DOI:** 10.1007/s00521-025-11039-2

**Published:** 2025-02-28

**Authors:** Jude Thaddeus Persia, Myung Kyun Sung, Soobum Lee, Devin E. Burns

**Affiliations:** 1 Department of Mechanical Engineering, University of Maryland, Baltimore County, 1000 Hilltop Circle, Baltimore, MD 21250 USA; 2 NASA Langley Research Center, Hampton, VA USA

**Keywords:** Topology optimization, Neural network, Postprocessing, Surrogate model, Wind tunnel balance, Parameterization

## Abstract

This paper proposes a general method of creating an accurate neural network-based surrogate model for postprocessing a topologically optimized structure. When topology optimization results are converted into computer-aided design (CAD) files with smooth boundaries for manufacturability, finite element method (FEM) based stresses often do not agree with the topology optimized results due to changes of surface and mesh density. The conversion between topology optimization derived results and CAD files often requires postprocessing, an additional fine tuning of the geometry parameters to reconcile the change of the stress values. In this work, a feedforward, deep artificial neural network (DANN) is presented with varying architecture parameters that are found for each stress output of interest. This network is trained with the data based on a combination of Design of Experiments (DoE) models that have the geometry dimensions as inputs and stress readings under various loads as the outputs. A DANN-based surrogate model is constructed to enable fine tuning of all relevant stress performance metrics. This method of constructing an artificial network-based surrogate model minimizes the number of FEM computations required to generate an optimized, post-processed design. We present a case study of postprocessing a wind tunnel balance, a measurement device that yields the six force and moment components of a test aircraft. It needs to be designed considering multiple stress measures under combinations of the six loading conditions. Excellent performance of a neural network is presented in this paper in terms of accurate prediction of the highly nonlinear stresses under combinations of the six loads. Von Mises stress predictions are within 10% and axial force sensor stress predictions are within 2% for the final post-processed topology. The results support its usefulness for postprocessing of topology optimized structures.

## Introduction

The use of topology optimization is expanding in many engineering fields. A topology optimized design distributes material across a specified design space to meet desired mechanical characteristics, such as stiffness or stress [1]. Flexures and/or compliant mechanisms have been designed using topology optimization (TO) to meet their load sensing and movement requirements. Richardson et al. [2] and Zhang et al. [3] both utilize robust topology optimization approach applied to the complaint mechanism and explicit definitions of beams in a truss structure. Lee & Gea also optimize a complaint mechanism design with respect to strain measurements, rather than stress [4]. However, the topology output is square element-based (or pixel-based) geometry rather than the vector-based geometry as seen in conventional computer-aided design (CAD) or finite element method (FEM) model construction. This results in a need for postprocessing into a model that can be parameterized by dimensions of individual features, such as flexures. Several different methods can be applied to convert the TO output to a vector-based model, while preserving the performance characteristics, such a CAD reconstruction initially using geometric approximations [1, 5]. Afterwards, identifiable flexures and beams are parameterized, and an additional optimization is conducted using the newly constructed CAD geometry. Bharanidaran & Ramesh have used this method on a compliant-based microgripper, where the reconstructed CAD model was used to identify trends in adjusting the individual parameters to approach the same performance levels produced in the TO process [6]. Topology optimized compliant mechanisms, especially those with geometric nonlinearity and with multiple degrees of freedom, often react nonlinearly from changes in flexure dimensions to stress behavior [7]. A neural network-based surrogate model is used to address this issue, showing greater accuracy in approximating the highly nonlinear behavior than classical regression [8]. Ktari & Elmansori outline the process of generating a network-based surrogate model to predict FEM results from a dataset of varying parameters [9]. This approach can be applied to geometric CAD models to predict stress results from changes in dimensions, as depicted by Gajewski et al. [10].

**Fig. 1:**
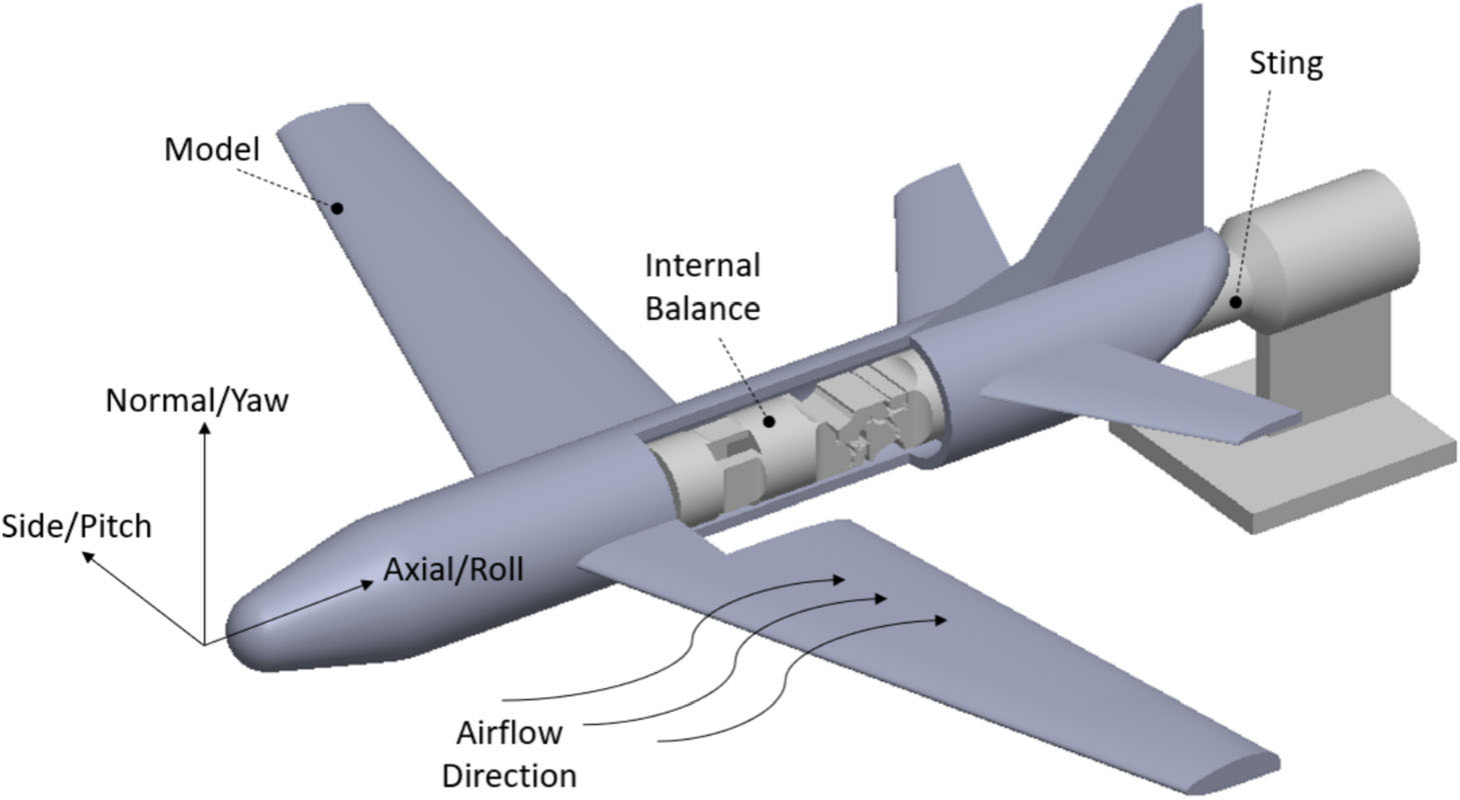
Aircraft model with internal balance

This paper considers these postprocessing approaches for the design of a force transducer mechanism. This mechanism is designed using topology optimization to be applied in a wind tunnel balance, a kind of six axis force sensor [11] used in the wind tunnel testing. When testing aircraft models in a wind tunnel, aerodynamic loads are measured to determine flight characteristics in a physical environment [12]. These loads are experienced in six components: axial force (**F**_a_), normal force (**F**_n_), side force (**F**_s_), pitching moment (**M**_p_), rolling moment (**M**_r_), and yawing moment (**M**_y_) and are measured using a force balance as shown in Fig. 1. Within the fuselage is an internal wind tunnel force balance. This is distinguished from an external balance, where the measurement components are located outside of the wind tunnel test section [13]. Internal balances incorporate the use of strain gauges placed in a Wheatstone bridge configuration to independently measure the six forces [1, 12]. This balance is affixed to the forward end of the model, while the rear section of the balance is coupled to the sting. The sting provides the rigid connection of the balance to a platform distanced from the model. When the model is under aerodynamic load, the forces and moments transfer from the airframe to the balance and generate bending deformation throughout the balance and its axial section [12]. The working principle of a balance is that strain gauges are placed along beams, or flexures, that are expected to elastically deform and generate strain only when under a specific load [14].

**Fig. 2:**
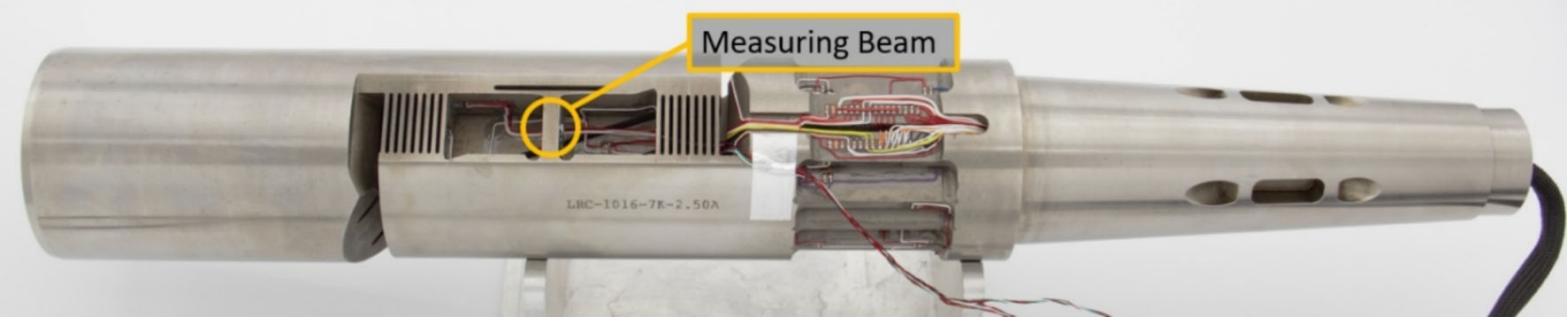
Conventional balance design

The axial load is of particular concern due to the nature of aircraft design: axial load, or aerodynamic drag, is inherently much lower than the other forces that maneuver most aircraft, such as an axial-to-normal force ratio as 1:8 considered in this work. Therefore, the axial section of a balance needs to be designed to generate the highest amount of response subject to axial load, while having little to no response subject to the other five loads [14]. Conventionally, the design includes many flexures that amplify the bending deformation of a singular beam, shown in Fig. 2 as the measuring beam. The load requirements result in a mechanically complex design that directs compliance to the measuring beam under axial force, while minimizing this compliance under the other forces by redistributing them around the beam. The geometric complexity with a large quantity of small cutouts throughout the axial section results in a longer lead time and price [15].

Topology optimization (TO) can be an alternative approach to design the balance (axial section). Topology-based geometry can limit the number of flexures and design features that need to be machined, and in this case, the TO can be formulated to project the entire machining area onto one plane. When the TO result is remodeled in conventional CAD or FEM, however, the TO results often do not agree with FEM results of the converted model due to changes in the surface and mesh density. The converted CAD geometry requires postprocessing, an additional fine tuning of the geometry parameters to compensate for changes of the stress values. This paper presents a neural network-based surrogate model that relates CAD geometry parameters and the stress-related performance measures for effective postprocessing of a topologically optimized wind tunnel balance. Surrogate model construction is focused on having an accurate estimation of multiple stress measures which are inaccurate in a cell-based topology optimization result. The neural network models are built per each stress measure and the accuracy is evaluated by comparing to the conventional response surface method.

The primary objective of this work is to minimize computation time of a design parameter optimization by using a regression model based on an artificial neural network. Specifically, this work uses the regression model for accurate and efficient postprocessing of topological optimization results. This involves training a neural network with sufficient data to have acceptable accuracy for use as a surrogate model for optimization. This work introduces a concept of combining several sampling schemes to take advantage of the properties unique to each scheme, such as rotatability or orthogonality. This allows for a broader exploration of the design space without requiring a substantially larger amount of data from using a full or fractional factorial with the same number of levels. This method produces a trained series of neural networks with high accuracy in model prediction and the network-based regression model finds optimal design parameters of an axial section to meet the design requirements.

This paper is organized as follows. Section 2 briefly introduces the design formulation and the output for the topology optimization. The structure for the artificial neural network used in this study is outlined in Sect. 3. Section 4 describes the transition from the topology output to a CAD-based format using a surrogate model. The methodology to locate sampling points, collect data, and construct the surrogate model is also described in this section. The result section presents postprocessing using this surrogate model. Lastly, the conclusion discusses the network-based accuracy and suggests future work.

## Topology optimization of axial section

The topology optimization (TO) of a wind tunnel balance (axial section) is briefly introduced in this section [1]. The TO problem for an axial section can be formulated to minimize von Mises stress under a combined loading case, subject to sensor stress constraints under various single-load cases. The TO formulation is presented as follows:

1 $$\max :V({\overline{\mathbf{x}}}^{(d)} )$$

2 $$s.t.:{\mathbf{Ku = f}}$$

3 $$\begin{array}{*{20}c} {:\sigma_{s} = \left| {\frac{{\sigma_{s1} \left( {{\overline{\mathbf{x}}}^{(d,i,e)} } \right) - \sigma_{s2} \left( {{\overline{\mathbf{x}}}^{(d,i,e)} } \right)}}{2}} \right| \ge \sigma_{0} } & {{\text{by}}\;{\mathbf{F}}_{a} } \\ \end{array}$$

4 $$\begin{array}{*{20}c} {:\sigma_{s} = \left| {\frac{{\sigma_{s1} \left( {{\overline{\mathbf{x}}}^{(d,i,e)} } \right) - \sigma_{s2} \left( {{\overline{\mathbf{x}}}^{(d,i,e)} } \right)}}{2}} \right| \le \alpha \sigma_{0} } & {{\text{by}}\;{\mathbf{F}}_{n} + {\mathbf{M}}_{n} } \\ \end{array}$$

5 $$\begin{array}{*{20}c} {:\sigma_{s} = \left| {\frac{{\sigma_{s1} \left( {{\overline{\mathbf{x}}}^{(d,i,e)} } \right) - \sigma_{s2} \left( {{\overline{\mathbf{x}}}^{(d,i,e)} } \right)}}{2}} \right| \le \alpha \sigma_{0} } & {{\text{by}}\;{\mathbf{M}}_{p} } \\ \end{array}$$

6 $$\begin{array}{*{20}c} {:\sigma_{\max } = c^{(d,i,e)} \sigma_{{{\text{pnorm}}}}^{(d,i,e)} \le \sigma_{\text{safe}} } & {{\text{by}}\;\sum {\mathbf{F}} } \\ \end{array},$$

where *V* is a volume of the design, and $\overline{\mathbf{x} }$ is a physical design variable from the projection method [16]. Superscripts *d*, *i*, and *e* represent dilated, intermediate, and eroded variations of the robust design, respectively. Since one of the driving factors in using a topology-based approach is reducing manufacturing complexity, the objective function is set to maximize the volume in the design domain to minimize the number of cuts in the machining process [17]. *σ*_s1_ and *σ*_s2_ in Eqs. 3–5 indicate the Y-directional stress from sensors 1 and 2. *σ*_0_ is the minimum sensor reading to satisfy the axial force sensing requirement under **F**_*a*_ in Eq. 3. The second and third constraints, Eqs. 4 and 5, are to suppress sensor readings by the non-axial load components (**F**_*n*_ + **M**_*n*_ and **M**_*p*_), where *α* is the restriction coefficient of 10% for the sensor performance. The last constraint in Eq. 6 is the safety constraint to limit the von Mises stress below the maximum allowed stress (*σ*_safe_) under the combined loading case (*ΣF* = **F**_*a*_ + **F**_*n*_ + **M**_*n*_ + **M**_*p*_). It is noted that only three force components are used in this study based on 2D design space – the final 3D design will be built by extruding the 2D geometry along *z* axis, so the stress outputs by the out of plane forces are minimal.

Equation 6 introduces P-norm (*σ*_pnorm_) which is used to evaluate the maximum von Mises stress (*σ*_max_). *c* is a normalization coefficient used to convert *σ*_pnorm_ to *σ*_max_. *σ*_pnorm_ is defined as:

7 $$\sigma_{{{\text{pnorm}}}} = \left[ {\sum\limits_{i = 1}^{n} {v_{i} \tilde{\sigma }_{i}^{Pn} } } \right]^{1/Pn},$$

where *n* is the number of elements in the design domain, subscript *i* is element index, *v*_*i*_ is elemental volume, and *Pn* is the norm parameter. Myung et al. determined *Pn* to be 2.5 for this TO problem [17]. The maximum von Mises of the analysis domain is represented by the P-norm stress with the infinity norm parameter (*Pn* = ∞) [18]. Stress singularity can occur when employing topology optimization and is resolved in this work using the relaxation method [19]. *σ*_*i*_ is marked with a tilde (~) to indicate a relaxed stress at element *i*. This stress evaluation is conducted per iteration of the TO to determine convergence. The TO in this study is based on the method of moving asymptotes (MMA) algorithm. A general flow of TO is shown below.

**Algorithm:**
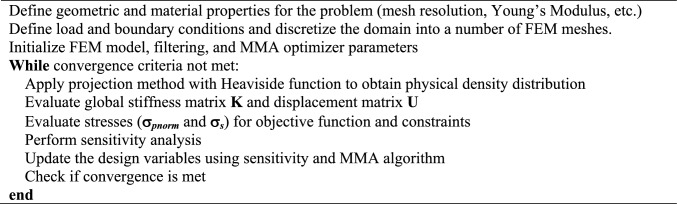
Topology Optimization using MMA

**Fig. 3:**
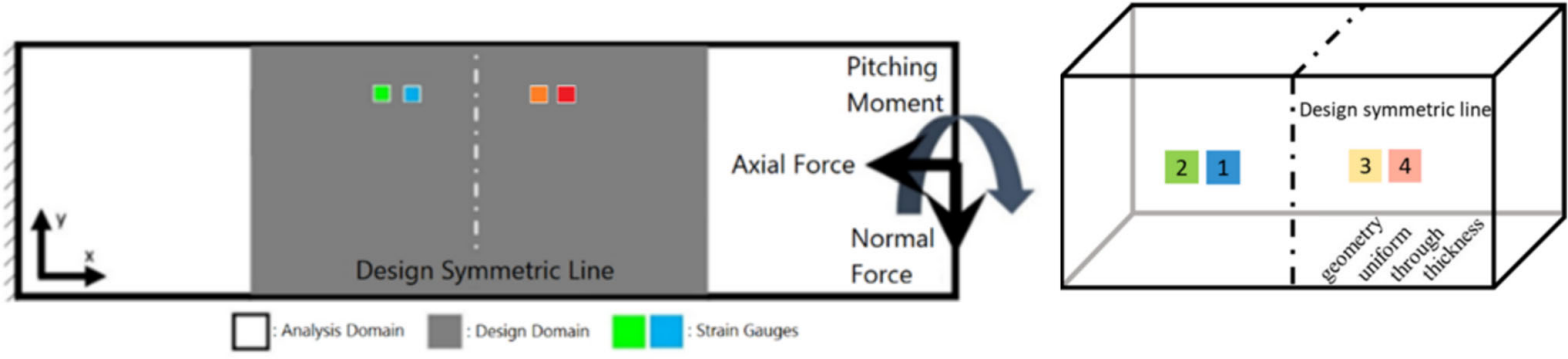
Analysis and design domain for topology optimization

The analysis and design domain of the topology optimization is shown in Fig. 3. The design space is 64mm long, while the overall length of the axial section is 148mm. Additional space outside of the design space is used to maintain the moment arm length of the applied loads. The sensor locations are highlighted in green and blue, with their mirrored counterparts in orange and red. The topology design utilizes a plane of symmetry normal to the axial direction, centered along the *x* axis of the design space.

**Fig. 4:**
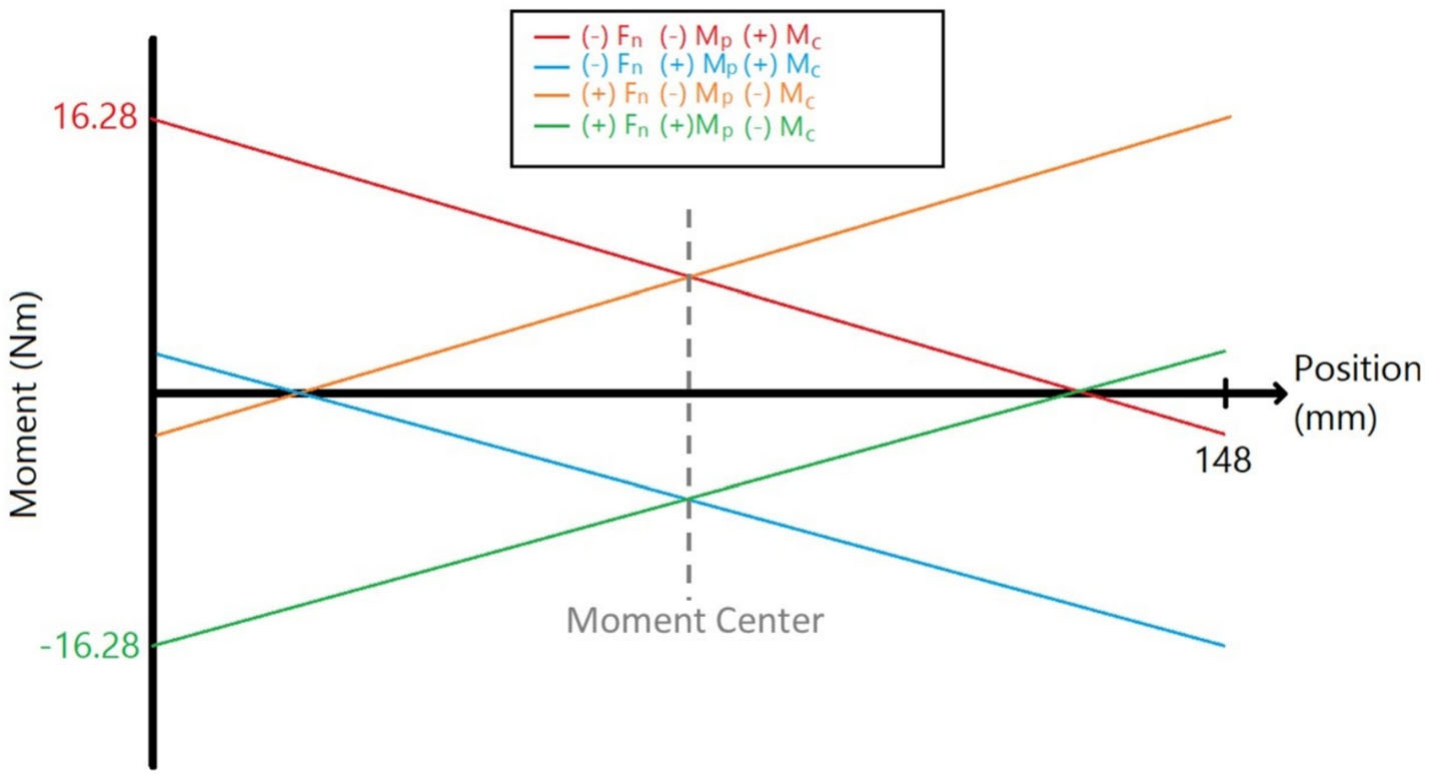
Moment diagrams under each loading scenario

The benefit of using this form of symmetry, when compared to the design in the previous study [5] is a balanced moment diagram across all orientations of non-axial loads. Figure 4 shows the details: the moment diagrams that consider the normal force (**F**_n_), counter moment (**M**_c_), and pitch moment (**M**_p_). **M**_c_ is utilized in the loading scenario to negate the moment by **F**_n_ at the center of the cantilever. From these four loading scenarios, the maximum moment is on either the far left or far right sides with a magnitude of 16.28 N-m. This indicates that as long as we keep design symmetry across a central *y* axis, the bending moment is measured symmetrically even though the directions of non-axial loadings change.

The axial section design is constructed using topology optimization, where stress performance characteristics are optimized using the design space shown in Fig. 3.

**Fig. 5:**
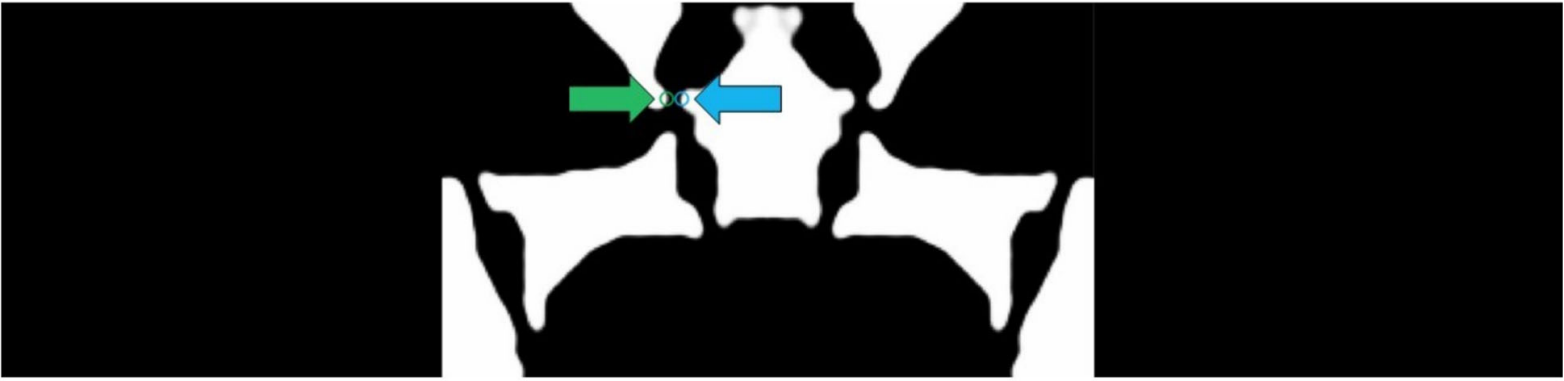
Topology optimization result, sensors highlighted in blue/green along measuring beam (Color figure online)

Figure 5 shows the optimized axial section as the result of TO. Narrow beam-like sections, or compliant flexures, are identified throughout the geometry. Under load, each of these flexures bend under elastic deformation that determines the corresponding bending stress and the sensor readings. The stresses are being measured at the sensor locations as highlighted in blue (sensor 1) and green (sensor 2).

The topology optimized result is based on a cell-based design approach and needs to be post-processed to have a valid performance even after converting to a smooth boundary geometry (e.g. CAD). Usually the stress measures—von Mises stresses, normal force stress, and pitching moment stress—are sensitive to the bending behavior of the flexure geometry. In the postprocessing, therefore, the flexure geometry parameters (e.g., length, thickness) are dimensioned with parameterization to allow for bending behavior to be controlled. This is necessary to allow a surrogate model to predict how each of the flexure parameters contributes to the stress measures of the structure [6]. This 2D TO image is the baseline to conduct the postprocessing optimization in the next section.

## Artificial neural network

Using a parameterized CAD model, one can build a surrogate model that can predict the response in output parameters from the change of inputs. One of the conventional approaches for building the surrogate model is the use of polynomial fitting with Response Surface Method (RSM). In this method, a regression model of each of the outputs is constructed separately using a number of data points based on a design of experiment (DoE) approach, so that the model can predict the overall stress behavior with any combination of input parameters [20].

Instead of creating regression models based on RSM in the form of *n*-th order polynomials, the DoE models can be analyzed through implementation of deep learning which uses multiple layers of neural networks to process data [21]. Deep learning methods vary in structure, with some examples including Convolutional Neural Network (CNN), Gated Recurrent Unit (GRU) and a Deep Artificial Neural Network (DANN).

Darwish utilizes CNN, GRU, and a combined CNN-GRU in a mix of categorical and numerical data for fitting a regression model for cutting tool wear estimation [22]. Data analysis using a Temporal Convolution Network (TCN) was also used in building a predictive model in an ion etch milling process [23]. These deep learning techniques are well-suited to problems with multiple data types, as well as those with time dependencies.

**Fig. 6:**
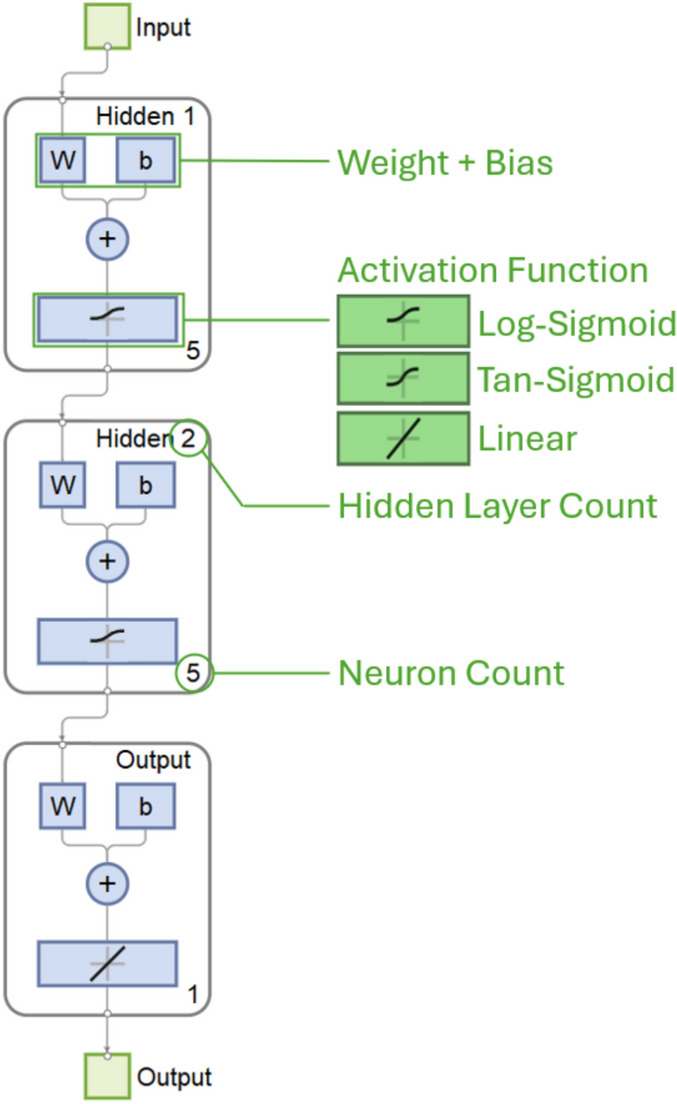
Neural network structure for two hidden layers

Since this postprocessing is intended to reduce the computational overhead of FEM evaluations, the dataset will be constructed with a relatively low quantity. For this work, a DANN is selected for the regression problem due to this data consisting of only a few hundred non-temporal numerical data. A standard feedforward artificial neural network (ANN) consists of an input layer, hidden layers, and output layer. Each layer consists of several neurons that determine which “path” a set of inputs should follow. The final output is a result of following the neural pathways of the network, where each step along the path is determined by an activation (transfer) function. A DANN is distinguished from standard ANN through its use of many interconnected hidden layers, generally more than two as shown in Fig. 6 [21, 24]. DANN is especially useful in problems with highly nonlinear responses, where standard RSM methods are poorly fitted [25]. A neural network-based model can also be used to capture nonlinear correlations in data that are not represented in classical mechanical beam theory [26]. A predictive model that has input–output relationships beyond a second order polynomial may not be effectively constructed through RSM, so a DANN method is used instead to have a greater level of accuracy in this paper.

Within the input layer, the inputs are normalized and processed through an activation function [21]. This study considers three different activation functions for each input, hidden, and output layer: linear, log-sigmoid, and hyperbolic tangent sigmoid shown in Eqs. 8–10, respectively.

8 $$f(n)= n$$

9 $$f(n)= \frac{1}{1+{e}^{-n}}$$

10 $$f(n)= \frac{2}{1+{e}^{-2n}}-1,$$

where *f* is the activation function output and *n* is the input value.

Each neuron contains a vector of weights *w* and a bias *b*, which are each determined during the neural network training and validation process. The procedure for processing input data through a trained neural network starts with the hidden layers, formulated as follows:

11 $$\overrightarrow{{h}_{j}}=\left\{\begin{array}{c}f\left({b}_{j}(1)+\sum_{i=1}^{N}{w}_{j}\left(1, i\right){*h}_{j-1}(i)\right)\\ \vdots \\ f\left({b}_{j}(M)+\sum_{i=1}^{N}{w}_{j}\left(M, i\right)*{h}_{j-1}(i)\right)\end{array}\right\},$$

where $\overrightarrow{{h}_{j}}$ is the $j$th hidden layer, $f(x)$ is the activation function in Eq. 2, ${b}_{j}$ is the $j$th bias vector, *M* is the number of neurons in that layer, *N* is the number of outputs from the previous layer, and ${w}_{j}$ is $j$th weight matrix. Each $\overrightarrow{h}$ is evaluated sequentially from the input layer to the output layer.

## Methodology

Using the 2D image generated by the topology optimization code, a FEM model is constructed as a 3D geometry of unit thickness of 1 mm [1]. The individual stress measurements at key locations across the design space are measured as the performance metrics. In detail, six Y-directional stress sensor performances at the measuring beam (two strain gauges attached side by side) under three loads (axial, normal, and pitch component loads) are measured by averaging stress along the strain gauge length on the measuring beam. Two sensors, where one is placed on each side, are expected to be used per measuring beam as shown previously in Fig. 5. Due to the symmetry condition of the model, only one of the measuring beams is used for the stress evaluation in each data point.

**Fig. 7:**
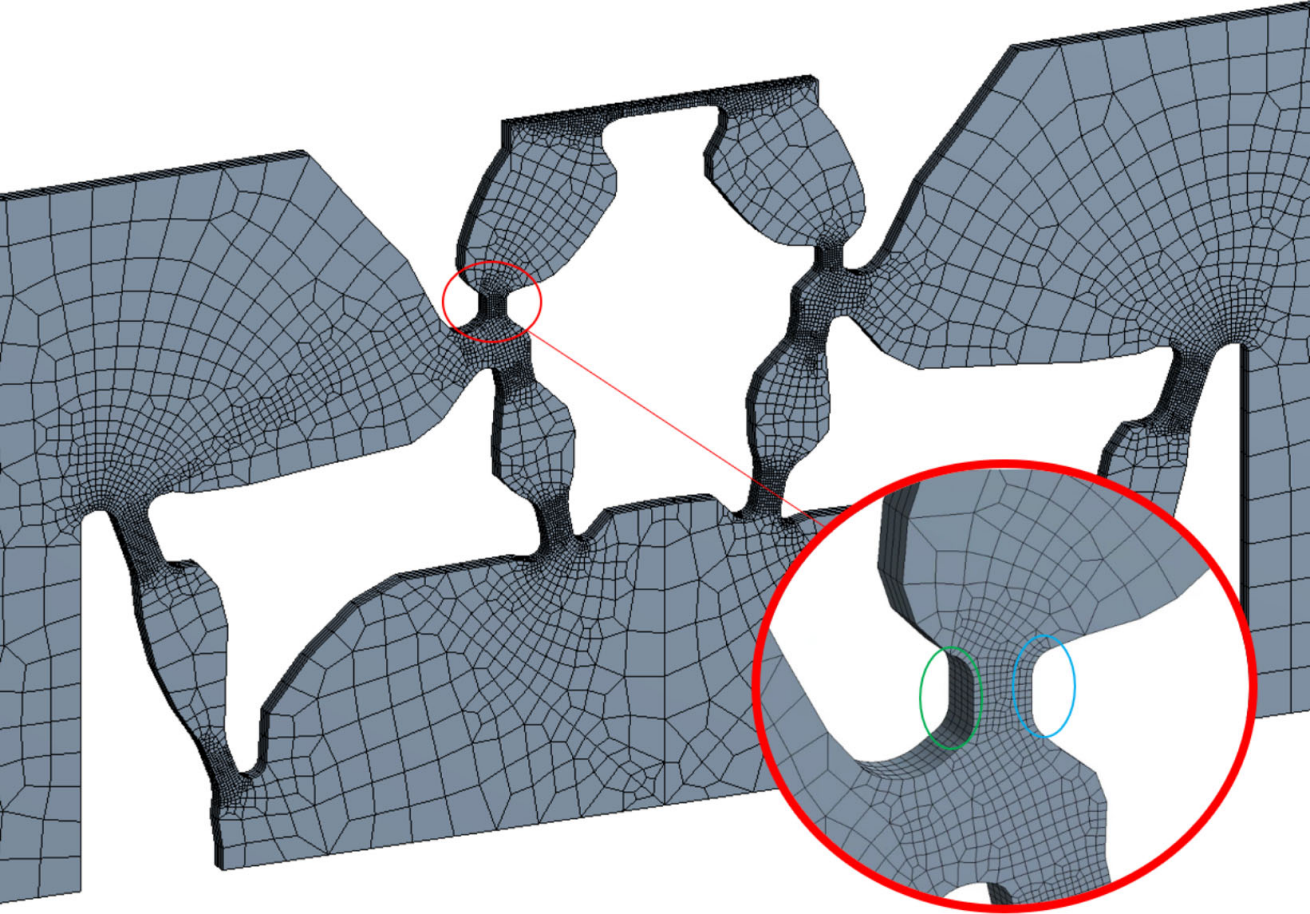
Mesh generation for axial section

The topology is generated with a uniform finite element size of 1/4 mm. A higher mesh density for select regions is considered for greater level of accuracy for FEM runs on ANSYS, shown in Fig. 7. Mesh elements are concentrated along flexures, and a coarse mesh is used for more rigid bodies. This mesh contains a total of 111,448 nodes and 21,872 s-order hexahedral elements.

**Fig. 8:**
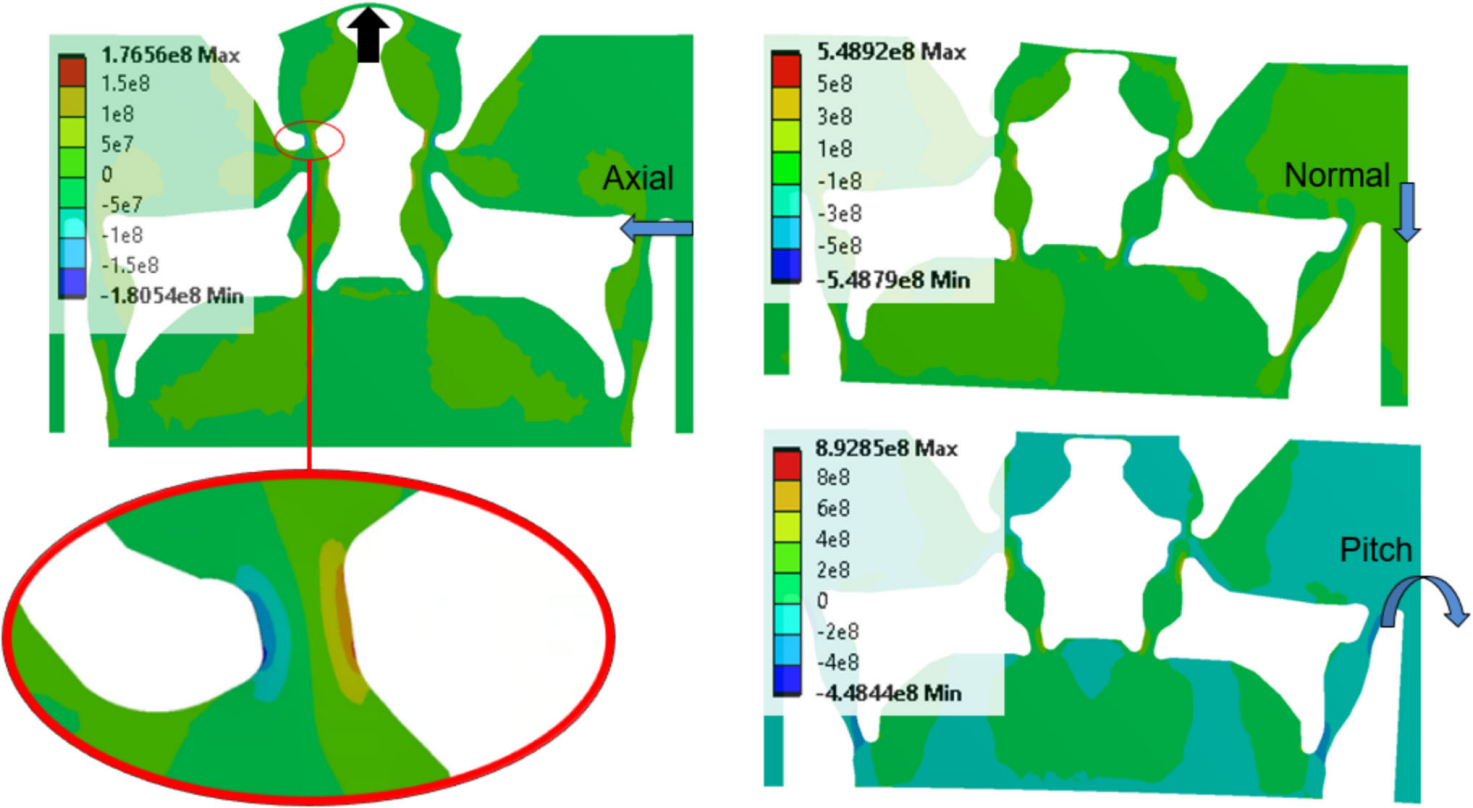
**a** Axial deformation (left), **b** pitch deformation (right), **c** normal deformation (center) and Y-directional normal stress (MPa)

FEM stresses are evaluated using this mesh, sharing the same load and boundary conditions as in the original TO problem definition. Under directional loads, the flexures generate stresses outlined by the deformation behavior shown in Fig. 8. Loading is represented by the arrow directions on the right side of each subfigure with the color gradients depicting Y-directional normal stresses in MPa under each corresponding load. The two large solid bodies outside of the design space (analysis domain in Fig. 3) are negligible in stress and excluded from the following figures. The loading conditions are still applied to the outermost edge of these bodies, and the arrows indicating the load orientation are only positioned to represent the loading type for each stress figure.

**Fig. 9:**
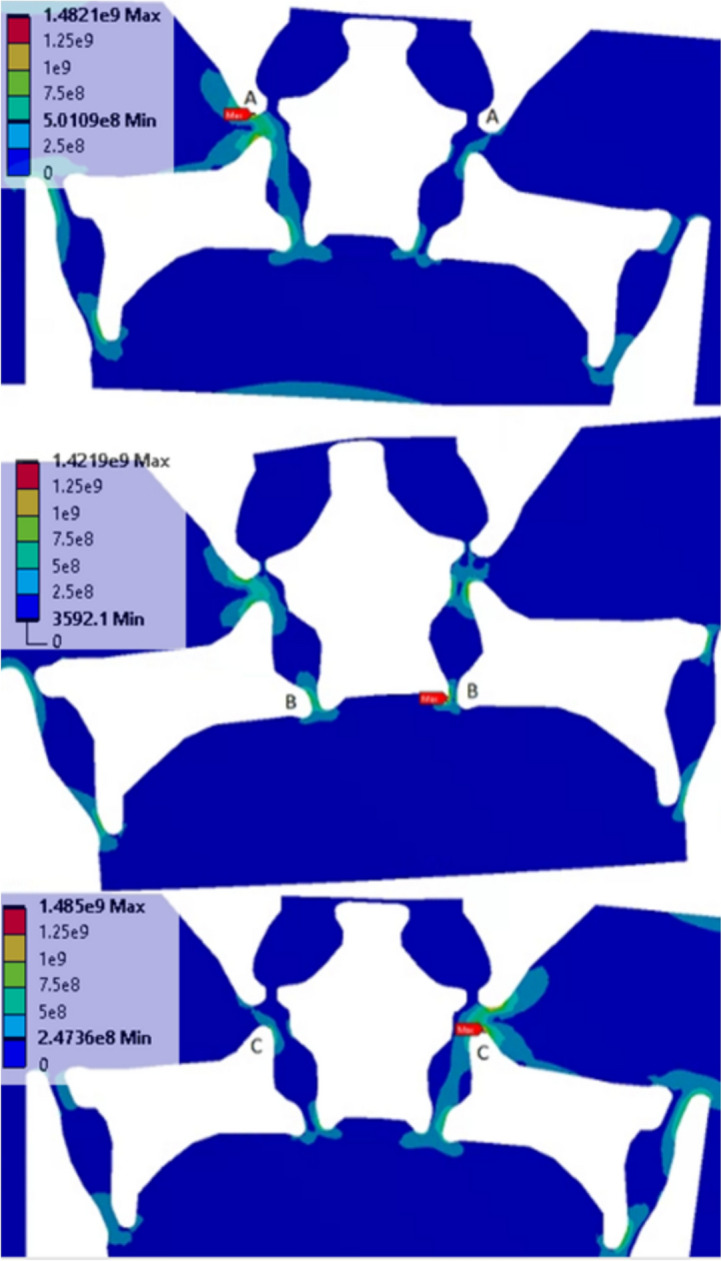
von Mises stress (MPa) peak locations

Figure 8a shows the greatest amount of bending deformation on the measuring beam under the axial loading as highlighted in red. The topmost flexure, indicated by the black arrow, bends (upside down U shape) to permit rotation of the rigid body directly above the measuring beam. However, this flexure bends in an S-shape under normal and pitch loads (Fig. 8b, c). Upon evaluation of von Mises stresses, it was found that the maximum von Mises stress is found among six locations between the four load combinations described in the previous section (Fig. 4). These locations are shown in Fig. 9, where the most prominent peak location is A, followed by C and B in terms of number of occurrences. These locations occur under cases, where flexure dimensions are along their boundaries across the four load orientation combinations.

From the six locations (A-A, B-B, and C-C) and the four load combinations (Fig. 4), twenty-four global von Mises stresses are potential output parameters. However, due to the symmetry of geometry and loading conditions, the overall case number of output von Mises stress is reduced by half (twelve). For example, when a peak stress occurs on the left A using standard normal and pitch load orientation (downward and clockwise), the same magnitude of stress occurs on the right A using a reversed normal load and pitch orientation (upward and counterclockwise). This property was observed across all locations and load orientation combinations because of geometric symmetry.

**Fig. 10:**
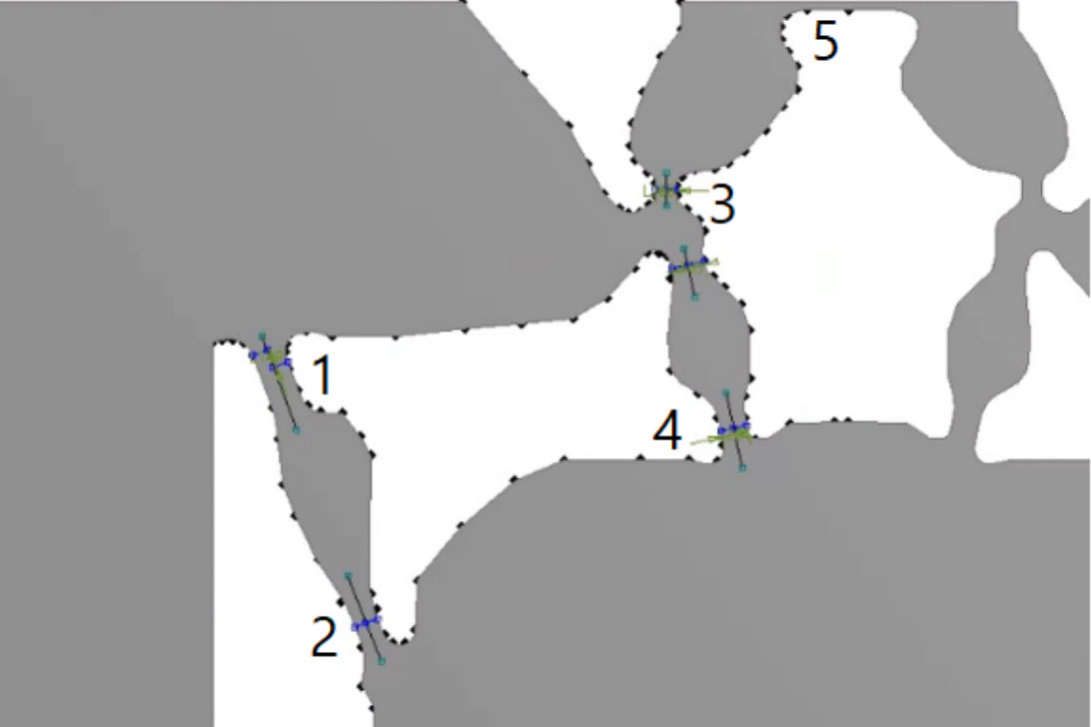
Flexure identification

Since this mesh is tied to CAD geometry, flexure dimensions are parameterized to allow for selective changes in stress responses in different areas. The postprocessing initially considers ten total input parameters as shown in Fig. 10. Five parameterized flexures are parameterized by length and thickness. These five were chosen due to their significant influence on the stress outputs relative to others. The measuring beam, flexure 3, is of greatest interest because it contains the points at which the sensor response (Y-directional stress components) is measured. Upon further FEM analysis of the model, lengths of hinges 2 and 4 were deemed inconsequential to the stress outputs and removed from the input parameter pool. The remaining eight inputs are used in the neural network and its performance is compared with a second order polynomial fitting RSM. This reduction of input parameters simplifies the surrogate model and requires fewer data points to construct a well-trained neural network. The remaining input parameters are then normalized and.

**Fig. 11:**
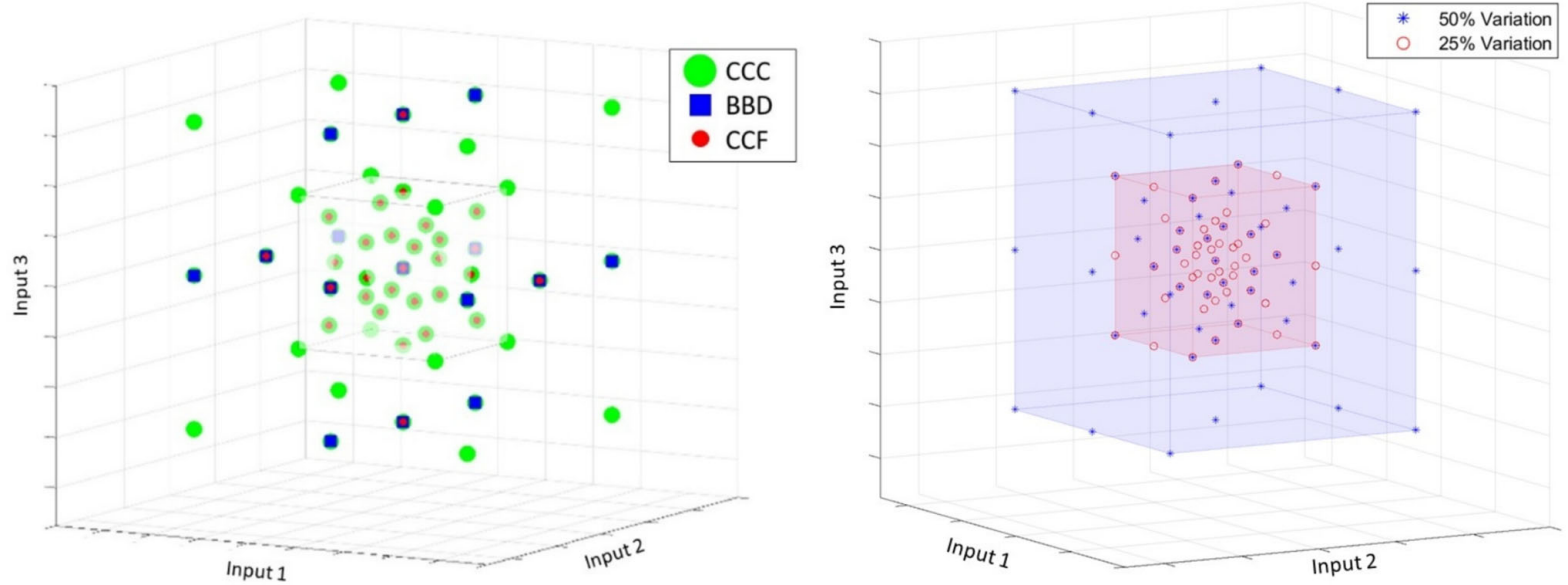
**a** Dataset DoE distribution (left), **b** complete dataset in 3D space (right)

For a balanced dataset generation and a high accuracy surrogate model, this study involves the use of several DoE types used in conjunction for dataset generation. Three designs of experiments are combined: Central Composite Circumscribed (CCC), Central Composite Face-Centered (CCF), and Box-Behnken Design (BBD) [25]. CCC, CCF, and BBD each have their own advantages when constructing a surrogate model in terms of having greater sensitivity to higher-order impacts [27, 28]. However, each individual DoE model is equally insufficient in predicting outputs when the model response is expected to be highly nonlinear, as is the case with the combined loading scenarios of the von Mises stress outputs. Rather than selecting one DoE model for training the dataset, this study uses all three in conjunction. This combination both maximizes the effectiveness of network training with a larger number of data points and mitigates the risk of insufficiently trained neurons [29]. This investigation deviates from similar studies [30–32] in generating ANN-based predictive models by combining several DoE models as a means of training a network with a larger number of input variables [30–32]. Figure 11a shows the points contributed by each DoE configuration in a 3D space, considering three input variables only for ease of visualization. The center point of the plot indicates the dimensions of the topology design before postprocessing. A reference cube halfway between center point and the boundaries (black solid line) is added as a visual aid to better discern the DoE structures within the densely populated central region. The BBD and CCF designs fill the boundaries and the midrange, while the CCC fills design points much closer to the origin. Each of overlapping points between CCC, CCF, and BBD is handled as a single data point in training.

Due to the high nonlinearity of the von Mises stress responses, the additional levels are crucial to constructing a well-fitted model. The dataset includes ± 25% maximum variation (red) and ± 50% maximum from the original geometric parameters (blue in Fig. 11b). As a result, six total DoE models are combined by the combination of the three DoE types (CCC, CCF, BBD) and the two boundary variations (25 and 50%), to complete the entire dataset. As with the overlapping points removed between DoE types, a combined count of 561 data points are created out of eight input parameters. This sampling strategy is well-balanced to account for nonlinear performance in each direction of design parameters. Appendix (Table 5) shows an example of the variations in dimensions using this method for only 3 input parameters. This combined dataset is then used to train a neural network for each output independently.

Each neural network utilized in MATLAB is structured as a feedforward, fully connected network using Levenberg–Marquardt back-propagation for use in regression modeling. This method is simple to implement and is an accurate method of modeling for FEM modeling problems [32]. The neural network within MATLAB is structured to output the predicted stress performance based on a combination of flexure dimensions. The network structure accounts for the six sensor outputs from directional loads (Axial, Normal, and Pitch) as well as eighteen von Mises stress outputs. Among the von Mises stresses, there are six distinct peak locations for each of the three highest load orientation combinations. Since each performance metric has its own network, the combined count of both sensor and von Mises outputs results in an overall network count of twenty-four. The *Z*-score method in Eq. 12 is applied to the stress outputs in the dataset, while the flexure dimensional inputs use their original values. The inputs are millimeter measurements within a very narrow range and thus did not require normalization.

12 $$z=\frac{x-\mu }{\sigma },$$

where $z$ is the normalized value for a particular input variable $x$, $\mu$ is the mean average of the data, and $\sigma$ is the standard deviation. The dataset (561 data points) is then divided for use with the neural network: 80% of the dataset is reserved for training, 10% for testing, and 10% for validating.

During construction of the networks for directional and von Mises stress, it was found that a single configuration of DANN with a fixed number of neurons and layers for each network was not capable of producing a model with reasonable agreement with the dataset. This was due to the extreme nonlinearity between von Mises peaks that occur at varying points along the topology, wherever certain flexures are minimized in stiffness. In contrast, the directional stress behavior is much more linear. In this study, various network hyperparameters are found for each stress output—number of layers, number of neurons per layer, and the type of hidden/output layer activation function (Eqs. 8–10) by maximizing Pearson correlation coefficient (*R*). When determining the optimal network architecture, a network’s correlation coefficient is assessed between the overall dataset and the model predictions using the following equation:

13 $$R=\frac{\text{cov}(A, B)}{{\sigma }_{A}{\sigma }_{B}}$$

This metric is defined as the covariance of A and B, which correspond to the arrays of trained network outputs and FEM outputs, divided by the standard deviation $\sigma$ of their respective data. A higher correlation coefficient correlates to greater accuracy in predicting FEM results within the dataset boundaries. This R is then used to assess each hyperparameter combination with a target value of 1. The range of number of layers is 2–10, while the number of neurons per layer was between 5 and 25. These ranges were selected based on iterative adjustments after each pass of all combinations [33]. In addition, the three activation function types (linear, log-sigmoid, and tangent sigmoid) are assessed for hidden layer and output layer, with each combination of layer and neuron count. This results in a total of 1701 hyperparameter combinations that are all tested per output parameter.

Network hyperparameters influence the accuracy of the regression model, and a specific parameter configuration can be better suited for some stress outputs than others. The network for each stress metric is trained with each possible configuration of network parameters outlined above. The stopping criterion for the number of epochs is fixed at a validation performance gradient of 1 × 10^−7^ and a performance goal of 0. The number of epochs is a maximum of 1000. This network training under Levenberg–Marquardt optimization minimizes Mean Square Error (MSE). In one network, the epoch with the lowest Mean Square Error (MSE) is used to compute the resulting weights and biases of that network [29]. Among these networks with different combinations of layer dimensions and activation functions, the hyperparameters producing the highest *R* are selected and used later in the surrogate model combining all networks.

Once an output parameter is tested and the arrangement of network hyperparameters that yield the highest *R* is selected, individual components of the network with that configuration (i.e., layer weights, bias, and normalization coefficients) are stored. The process of generating and storing the network components is repeated for each stress metric. Once each output has its network object components stored, each set of weights, biases, and coefficients are used in parallel as an interconnected neural network. This network combination accepts an input parameter array and outputs all the stress outputs at the same time as a surrogate model. This array includes the geometric flexure parameters of the topology.

The network objects for each output are used in conjunction to construct the surrogate model that considers all outputs at the same time. The interconnected neural network is then optimized with an interior-point least squares regression algorithm to obtain the global minimum of von Mises stress, while satisfying the sensor constraints [34, 35].

## Results

To train neural networks using raw FEM-generated stress data, a normalization scheme is applied to the stress values for each of the networks. Von Mises stresses are generally several orders of magnitude higher than the directional load stresses due to the nature of the combined load scenarios. The complete dataset was used to evaluate each combination of network parameters for each neural network to define the architectures shown in Table 1.

**Table 1:** Network Architectures

	Output Parameter	*R*	Layer count	Neuron count	Hidden Activation	Output Activation
Axial sensor stress	Axial: Left	0.9999	3	12	logsig	purelin
	Axial: Right	0.9998	2	9	tansig	purelin
	Normal: Left	0.9994	2	16	logsig	tansig
	Normal: Right	0.9995	2	8	logsig	tansig
	Pitch: Left	0.9998	2	16	logsig	purelin
	Pitch: Right	0.9998	2	14	tansig	purelin
von Mises stress	Original Load Orientation—A	0.9855	10	19	tansig	tansig
	Original Load Orientation—B	0.9998	2	7	tansig	tansig
	Original Load Orientation—C	0.921	6	14	logsig	purelin
	Reverse Normal Force—A	0.9519	3	13	logsig	tansig
	Reverse Normal Force—B	0.9996	3	6	logsig	purelin
	Reverse Normal Force—C	0.9714	6	22	tansig	tansig
	Reverse Pitch Moment—A	0.9645	8	12	logsig	purelin
	Reverse Pitch Moment—B	0.9996	2	9	tansig	purelin
	Reverse Pitch Moment—C	0.9797	8	16	tansig	tansig
	Reverse Normal Force and Pitch Moment—A	0.9819	6	8	tansig	purelin
	Reverse Normal Force and Pitch Moment—B	0.9998	4	8	logsig	purelin
	Reverse Normal Force and Pitch Moment—C	0.9022	6	17	logsig	tansig

In conventional force balance nomenclature, the single directional load outputs are expressed with respect to the axial sensor. The three directional loads are applied, and the output Y-directional stresses are measured on both left and right sides. Each of the networks have a correlation coefficient above 0.9 as shown in Table 1. The directional load stresses have the greatest correlation, due to the more linear relationship of stress with a single axis load (“sensor stress” in Table 1). It is noted that the number of layers is consistently along the minimum value of two, with only one exception where the layer count was just three. The combined load scenarios are more nonlinear in response (“von Mises stress” in Table 1), which result in a slightly lower correlation between the flexure dimensions and stress outputs, while also showing a much wider range of network architectures to maximize the correlation (especially in terms of number of neurons). Von Mises at location B has a more linear response than the other two location pairs, and as a result has a much higher correlation coefficient. This can be further shown in Table 6 in the appendix section, which outlines additional performance metrics of the network models.

**Fig. 12:**
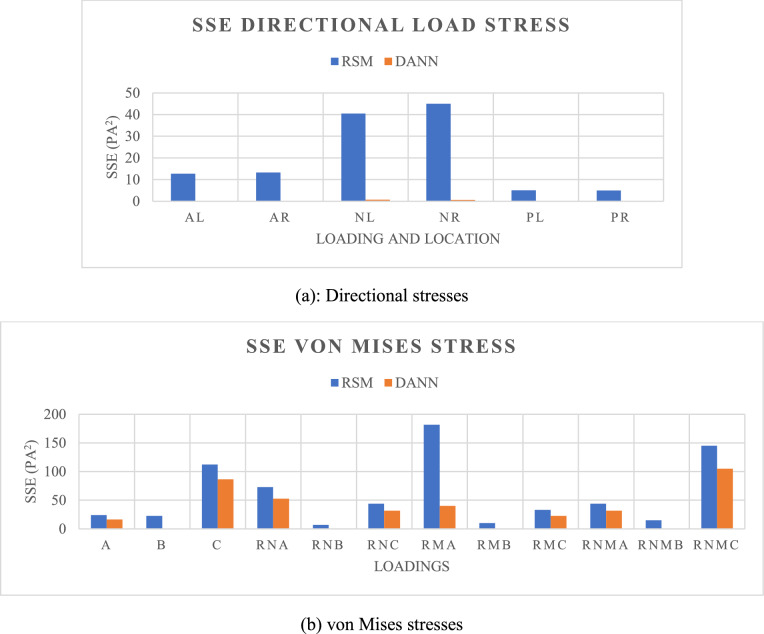
Sum of the squared error

The accuracy of the networks is compared to a standard second order RSM method using the same datasets and normalization factors. The RSM model for each output is described by the following the second order model [20]:

14 $$y={\beta }_{0}+\sum_{i=1}^{k}{\beta }_{i}{x}_{i}+\sum_{i=1}^{k}{\beta }_{i}{{x}_{i}}^{2}+\sum_{i<j}\sum {\beta }_{ij}{x}_{i}{x}_{j}+\epsilon,$$

where weight $\beta$ and bias $\epsilon$ factors are determined. The goodness of fit for this model is compared to that of the DANN in Table 2 using the sum of the squared error (SSE) as shown in Eq. 15:

15 $$\text{SSE}= \sum_{i=1}^{k}{\left(\widehat{{y}_{i}}-{y}_{i}\right)}^{2},$$

where $k$ is the total number of data points, ${y}_{i}$ is the numerical output from FEM determined at data point *I*, and $\widehat{{y}_{i}}$ is the surrogate model prediction with the same inputs. The error values for each of the individual networks are lower when using the DANN model in all cases. Some outputs with high nonlinearity, such as the von Mises stress at location C, have substantially high error but are still lower when using the network model. A graphical representation of the difference in error is shown in Fig. 12 of which the *x* axis label can be shown in Table 2. To verify that this arrangement of DoE produces sufficient data to train the regression models for accurate prediction, the same quantity of data is produced using a D-optimal (one of the popular classical forms of DoE) and compared as presented in Appendix.

**Table 2:** SSE Values for each method

	Output Parameter	RSM – 2nd Order	DANN
Axial sensor stress	Axial: Left	12.669	0.137
	Axial: Right	13.241	0.201
	Normal: Left	40.437	0.708
	Normal: Right	44.971	0.572
	Pitch: Left	5.010	0.214
	Pitch: Right	4.928	0.203
von Mises stress	Original Load Orientation—A	23.930	16.290
	Original Load Orientation—B	22.673	0.255
	Original Load Orientation—C	112.215	86.430
	Reverse Normal Force—A	72.936	52.701
	Reverse Normal Force—B	6.941	0.441
	Reverse Normal Force—C	43.958	31.554
	Reverse Pitch Moment—A	181.658	39.965
	Reverse Pitch Moment—B	9.893	0.403
	Reverse Pitch Moment—C	33.141	22.725
	Reverse Normal and Pitch Loads—A	43.958	31.554
	Reverse Normal and Pitch Loads—B	14.955	0.255
	Reverse Normal and Pitch Loads—C	145.029	105.023

**Fig. 13:**
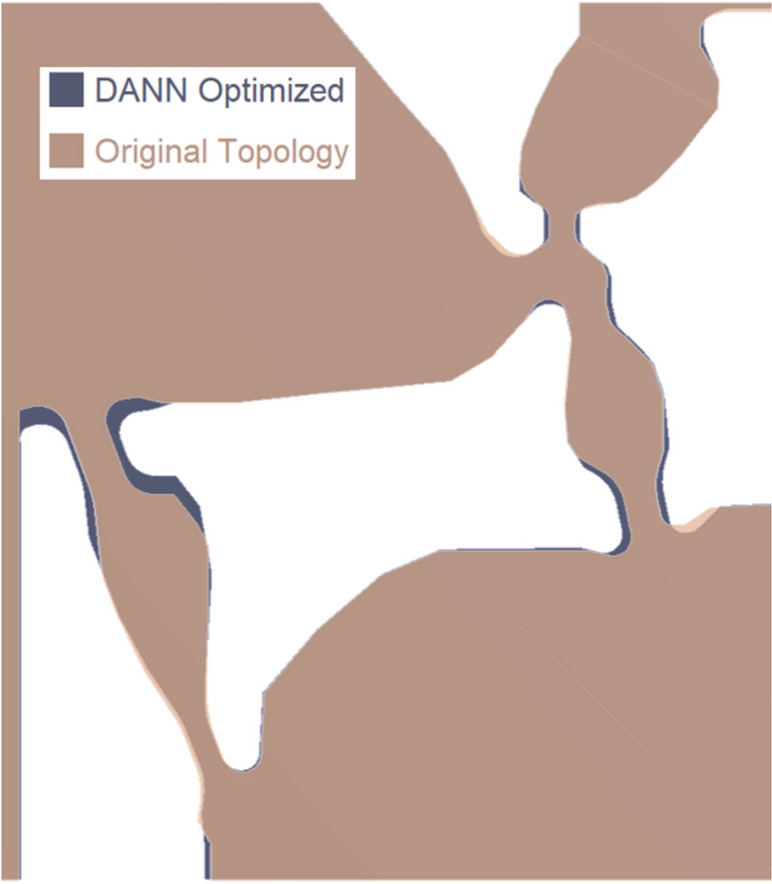
Optimized Structure comparison

Using the DANN surrogate model, the von Mises maximum stress is minimized as the objective function, and sensor evaluations are used as the constraints. The constrained optimization formulation outlined in Eqs. 16–20 is used for postprocessing:

16 $$\text{min}:{\sigma }_{\text{max}}\quad\text{ by}\; \sum {\varvec{F}}$$

17 $$s.t. :{\sigma }_{s1}-{\sigma }_{s2}\le {\sigma }_{0}\quad \text{by}\; {{\varvec{F}}}_{\text{normal}+\text{counter}}$$

18 $$:{\sigma }_{s1}-{\sigma }_{s2}\le {\sigma }_{0}\quad \text{by}\; {{\varvec{F}}}_{\text{pitch}}$$

19 $$:{\sigma }_{s1}\ge {\sigma }_{A} \quad\text{by}\; {{\varvec{F}}}_{\text{axial}}$$

20 $$:{\sigma }_{s2}\le -{\sigma }_{A}\quad \text{by}\;{{\varvec{F}}}_{\text{axial}},$$

where ${\sigma }_{\max}$ is the maximum von Mises stress among the array of von Mises stresses from the three locations. ${\sigma }_{s1}$ and ${\sigma }_{s2}$ are the sensor readings on the measuring beam, where 1 is the left side and 2 is the right side according to Fig. 8. ${\sigma }_{0}$ is the Y-direction stress reading of 15 MPa under normal force and pitch load (effectively an interaction of normal and pitch on the axial force bridge). ${\sigma }_{A}$ is the target axial load Y-direction stress reading of 75 MPa. MATLAB R2022b is used on a system using an AMD Ryzen 5800X CPU on Windows 10. The MATLAB optimization using this formulation resulted in the DANN-optimized output shown in Tables 3 and 4. Elapsed time for optimization is 42 s after 311 iterations and 3000 function evaluations. The comparison in hinge parameters before and after optimization is also shown in Fig. 13.

**Table 3:** DANN Optimization Inputs (mm)*

Parameters	Original (mm)	DANN (mm)	Δ*T*/*L* (%)
L1	2.847	1.423	175.9
T1	1.593	2.197	
*L2	1.512	1.512	− 19.36
T2	1.224	0.987	
L3	0.604	0.906	− 5.91
T3	1.072	1.513	
*L4	1.2	1.2	50.0
T4	1.39	2.085	
L5	2.165	1.988	− 40.67
T5	0.413	0.225	

*Parameters not meaningfully affects the stresses

**Table 4:** DANN optimization results and verification

	Constraints	DANN (MPa)	Target (MPa)	Percent error (%)
Axial sensor stress	Axial: Left	− 75.0	− 76.0	1.29
	Axial: Right	75.0	73.6	1.97
	Normal: Left	− 24.9	− 21.0	18.42
	Normal: Right	5.1	6.9	26.18
	Pitch: Left	5.2	16.2	67.65
	Pitch: Right	− 24.8	− 23.4	5.81
von Mises stress	Original Load Orientation—A	1373.0	1346.0	2.01
	Original Load Orientation—B	512.7	527.2	2.75
	Original Load Orientation—C	1358.0	1355.7	0.17
	Reverse Normal Force—A	371.5	360.9	2.93
	Reverse Normal Force—B	615.0	614.8	0.03
	Reverse Normal Force—C	612.9	673.3	8.97
	Reverse Pitch Moment—A	1323.8	1326.6	0.21
	Reverse Pitch Moment—B	616.2	631.8	2.47
	Reverse Pitch Moment—C	1281.0	1308.6	2.11
	Reverse Normal Force and Pitch Moment—A	362.1	347.8	4.11
	Reverse Normal Force and Pitch Moment—B	794.7	787.5	0.92
	Reverse Normal Force and Pitch Moment—C	878.1	858.0	2.34

Beam stiffness *F/δ*, as expressed by Eq. 21, is cubically proportional to *T*/*L* and is shown in Table 4. The last column indicates the percent change in ratio of beam thickness to length from the original geometry to the DANN-optimized geometry. Hinges 1 and 4 are increased in stiffness, while hinges 2, 3, and 5 are reduced. As shown previously in the axial force deflection in Fig. 8, hinge 5 is directly involved in rotating the rigid body linked to the upper end of hinge 3, the sensor hinge, and is reduced in stiffness for directional load sensitivity. Hinge 2 manipulates the amount of axial deformation to the lower end of the hinge 3 and is also reduced in stiffness. Hinges 1 and 4 are stiffened to reduce von Mises stress at location B, shown previously by the comparison in von Mises stresses in Fig. 9. Figure 13 shows the visual changes in beam dimensions throughout the geometry before and after DANN optimization.

21 $$\frac{F}{\delta }=\frac{Eb}{4}{(T/L)}^{3}$$

Between the FEM-verified ANSYS values and DANN model outputs shown in Table 4, von Mises stress outputs are within 10% of each other. It is also noted that the higher magnitude outputs above 1GPa have lower error of below 3%. The larger percent errors seen in the directional stresses for normal and pitch are attributed to the low relative stress levels (< 25 MPa) of the normal and pitch loads on the axial bridge. These two stress measurements also change signs throughout the dataset due to some hinge dimension combinations switching the measuring beam’s bending direction under load. However, the DANN predicted stresses themselves are only erroneous by 11 MPa at the highest.

## Conclusion

A method of constructing a neural network-based surrogate model is suggested in this paper for general postprocessing of the topology optimized geometry. This surrogate model was used to determine the optimal axial section flexure dimensions after postprocessing the original TO result into a configurable CAD model. Training the network with several hundred data points results in networks with sufficiently high correlation and small errors for use in predicting stress values at interpolated points between the dimension boundaries. The DANN model is shown to have lower error compared to a conventional second order RSM model to create a surrogate model using the same data. Our previous work is also augmented by this model through a more streamlined postprocessing approach, reducing the computational time with the surrogate model rather than manual adjustments and repeated RSM modeling directly applied to the FEM model. A key contribution of this work is the integration of various DoE techniques which allow for a robust training dataset that addresses the challenges of nonlinearity in stress behaviors. This approach streamlines the postprocessing phase of topology optimization, reducing the reliance on iterative finite element evaluations. The result of this work is a method of generating a neural network-based surrogate model for postprocessing complaint structures designed using topology optimization.

Several future works are suggested in terms of neural network modeling, program layout update, and handling 3D models. First, from a neural network architecture point of view, the current method involves evaluations of every combination of network hyperparameters within a specified range, rather than a heuristic approach based on recorded trends between hyperparameters and correlation coefficients. Since the number of neurons and layers are numerical, these can be directly mapped to trends in Pearson correlation coefficient to prevent an exhaustive search for the ideal hyperparameter selection. This study also exclusively uses DANN for machine learning. Extensions of ANN and other machine learning methods will be considered in future work. Currently our method is also limited in the range of DoE types studied in the dataset construction. Randomized sequential data sampling involving other types of DoEs such as Latin hypercube may be tested and compared. Second, our method also heavily involves the use of commercial FEM software for the postprocessing stress evaluations, which requires manual data transfer between programs. Future work can involve exporting the initial CAD-based FEM mesh into MATLAB to directly manipulate the FEM model for direct data acquisition and seamless process to train the networks. Third, handling 3D wind tunnel balance designs are required to investigate more flexible design update in a higher dimension of design space, and apply to other force measurement systems.

## Data Availability

All data generated or analyzed during this study are included in this article and in the online repository. Derived data supporting the findings of this study are available from the corresponding author upon request. The code has been released online: https://github.com/jtpersia/DANN_TO.

## Appendix

### Supplementary data for the combined DoE

Table

**Table 5:** Dataset example with three variables

L3	T3	T4
0.604	1.072	1.390052
0.453	1.072	1.390052
0.5285	1.072	1.390052
0.755	1.072	1.390052
0.6795	1.072	1.390052
0.604	0.804	1.390052
0.604	0.938	1.390052
0.604	1.34	1.390052
0.604	1.206	1.390052
0.604	1.072	1.042539
0.604	1.072	1.216296
0.604	1.072	1.737565
0.604	1.072	1.563809
0.302	1.072	1.390052
0.906	1.072	1.390052
0.604	0.536	1.390052
0.604	1.608	1.390052
0.604	1.072	0.695026
0.604	1.072	2.085079

^5^ outlines several of the data points constructed using the combined DoE types (Fig. 11) for making a partial 5-level design. This table includes three variables: length of hinge 3 (L3), thickness of hinge 3 (T3) and thickness of hinge 4 (T4). All the variations of the three are present in the complete dataset of 8 variables.

Table

**Table 6:** Network architecture additional performances (Combined DoE)

	Output Parameter	Epochs	Mean-Square Error (z-score normalized)
Axial sensor stress	Axial: Left	65	1.73e−5
	Axial: Right	35	1.23e−4
	Normal: Left	39	2.16e−5
	Normal: Right	52	1.29e−4
	Pitch: Left	41	7.66e−6
	Pitch: Right	42	1.94e−6
von Mises stress	Original Load Orientation—A	18	1.20e−2
	Original Load Orientation—B	44	2.47e−4
	Original Load Orientation—C	16	5.18e−2
	Reverse Normal Force—A	21	2.49e−2
	Reverse Normal Force—B	95	4.51e−4
	Reverse Normal Force—C	16	2.14e−2
	Reverse Pitch Moment—A	27	4.06e−2
	Reverse Pitch Moment—B	59	4.39e−4
	Reverse Pitch Moment—C	15	1.59e−2
	Reverse Normal Force and Pitch Moment—A	27	1.92e−2
	Reverse Normal Force and Pitch Moment—B	37	2.35e−4
	Reverse Normal Force and Pitch Moment—C	16	2.9e−2

^6^ contains additional metrics for each independent neural network prior to combining them to create the surrogate model. The number of epochs, or training cycles using the complete dataset, are counted alongside the mean-square error (MSE). MSE is unitless due to being calculated from normalized data points with the z-score method.

### Comparison to D-optimal sampling

To verify that the proposed DoE produces sufficient data to train the regression models for accurate prediction, the same quantity of data is produced using a D-optimal (one of the popular classical forms of DoE) and compared. Due to the nonlinearity of the stress responses under multiple loads, the 25% boundary was selected for its smaller increment size to improve model accuracy. This D-Optimal dataset is a 3-level design using the 25% boundaries and a center point. The MATLAB function “cordexch” was used to produce a coordinate-exchange D-Optimal design matrix used in this study.

Table

**Table 7:** Network architectures (D-Optimal)

	Output Parameter	R	Layer count	Neuron count	Hidden Activation	Output Activation
Axial sensor stress	Axial: Left	0.7178	6	8	tansig	purelin
	Axial: Right	0.742	4	16	tansig	purelin
	Normal: Left	0.7078	5	12	tansig	purelin
	Normal: Right	0.7841	7	13	tansig	tansig
	Pitch: Left	0.6918	7	14	logsig	purelin
	Pitch: Right	0.7417	6	15	logsig	purelin
von Mises stress	Original Load Orientation—A	0.7052	5	10	tansig	tansig
	Original Load Orientation—B	0.7189	3	14	logsig	tansig
	Original Load Orientation—C	0.7125	5	15	tansig	tansig
	Reverse Normal Force—A	0.714	5	15	tansig	purelin
	Reverse Normal Force—B	0.709	3	14	logsig	tansig
	Reverse Normal Force—C	0.6799	6	16	tansig	purelin
	Reverse Pitch Moment—A	0.6474	3	9	tansig	tansig
	Reverse Pitch Moment—B	0.7133	4	15	logsig	purelin
	Reverse Pitch Moment—C	0.6912	5	15	logsig	purelin
	Reverse Normal Force and Pitch Moment—A	0.7011	5	11	logsig	tansig
	Reverse Normal Force and Pitch Moment—B	0.6794	3	16	tansig	tansig
	Reverse Normal Force and Pitch Moment—C	0.6829	2	9	logsig	purelin

7 contains the network hyperparameters for the D-Optimal-based DANN that produce the highest *R*. The Pearson coefficients are consistently between 0.64 and 0.78, indicating a poor fit to the dataset compared to the combined DoE producing R values consistently above 0.9 (Table 1).

Table

**Table 8:** SSE Values for each method

	Output Parameter	RSM–2nd Order	DANN
Axial sensor stress	Axial: Left	403.0981	276.0623
	Axial: Right	403.3191	267.0518
	Normal: Left	387.0805	286.8246
	Normal: Right	392.0051	219.3256
	Pitch: Left	394.6101	292.2368
	Pitch: Right	397.0055	382.9627
von Mises stress	Original Load Orientation—A	421.129	283.6259
	Original Load Orientation—B	411.2335	280.2715
	Original Load Orientation—C	446.0955	277.0962
	Reverse Normal Force—A	423.7114	276.1794
	Reverse Normal Force—B	411.7483	281.6865
	Reverse Normal Force—C	441.9855	301.9428
	Reverse Pitch Moment—A	423.1234	332.2023
	Reverse Pitch Moment—B	407.9195	275.5786
	Reverse Pitch Moment—C	449.9433	295.436
	Reverse Normal and Pitch Loads- A	422.6194	285.9754
	Reverse Normal and Pitch Loads—B	407.7602	305.684
	Reverse Normal and Pitch Loads—C	448.7625	310.057

^8^ contains the SSE for the RSM and DANN regression models fitted to the D-Optimal dataset. The errors are considerably higher than those of the combined DoE in both cases (Table 2). Most notably, the DANN still has lower error in all cases with this dataset.
